# Evaluation of perineal muscle strength in the first trimester of pregnancy^1^

**DOI:** 10.1590/0104-1169.3600.2492

**Published:** 2014

**Authors:** Adriana de Souza Caroci, Maria Luiza Gonzalez Riesco, Bianca Moraes Camargo Rocha, Letícia de Jesus Ventura, Sheyla Guimarães Oliveira

**Affiliations:** 2 PhD, Professor, Escola de Artes, Ciências e Humanidades, Universidade de São Paulo, São Paulo, SP, Brazil; 3 PhD, Associate Professor, Escola de Enfermagem, Universidade de São Paulo, São Paulo, SP, Brazil; 4 Midwife; 5 Midwife, Hospital Geral de São Mateus Dr. Manoel Bifulco, São Mateus, SP, Brazil; 6 RN, Pronto Socorro e Maternidade Municipal Zoraide Eva das Dores, Itapecerica da Serra, SP, Brazil

**Keywords:** Pelvic Floor, Muscle Strength, Pregnancy, Urinary Incontinence, Pregnancy Trimester, First

## Abstract

**OBJECTIVES:**

to analyze the Pelvic Floor Muscle Strength (PFMS) of pregnant women with one or more vaginal or cesarean deliveries; to compare the PFMS of these with pregnant women with the PFMS of primiparous women.

**METHODS:**

cross-sectional study with women up to 12 weeks pregnant, performed in Itapecerica da Serra, São Paulo state, from December 2012 to May 2013. The sample consisted of 110 pregnant women with one or more vaginal deliveries or cesarean sections and 110 primigravidae. The PFMS was evaluated by perineometry (Peritron(tm)) and vaginal digital palpation (modified Oxford scale).

**RESULTS:**

the average PFMS in pregnant women with a history of vaginal delivery or cesarean section was 33.4 (SD=21.2) cmH2O. From the Oxford scale, 75.4% of the pregnant women with previous vaginal or cesarean deliveries presented grade ≤ 2, and 5.5% grade ≥ 4; among the primiparae, 39.9% presented grade ≤ 2, and 50.9% grade ≥ 4, with a statistically significant difference (p<0.001). From the perineometry, there was no statistically significant difference between the PFMS and age, type of delivery, parity, body mass index, and genitourinary tract symptoms, however, there was a statistically significant difference between the pregnant women with and without a history of episiotomy (p=0.04). In the palpation, none of the variables showed a statistically significant difference.

**CONCLUSION:**

pregnancy and childbirth can reduce the PFMS.

## Introduction

The pregnancy, type of delivery, perineal conditions, and parity may influence the pelvic floor muscle strength (PFMS), causing morbidities of the genitourinary urinary tract and a negative effect in relation to the sexual, physical, psychological, and social health of the woman^(^ 1 ^-^ 2 ^)^.

A study that evaluated the quality of life in 77 women with urinary incontinence (UI) 90 days after childbirth identified the most common symptoms as: micturition frequency (88.3%), nocturia (87%) and urge incontinence (54.5%). The authors concluded that although the loss of urine was small, it was frequent and caused an impact in the life of the women, interfering significantly in their physical and mental health^(^ 3 ^)^.

Regarding parity, a cohort study identified an increased prevalence of UI that remained for one year after delivery, among primiparae, compared to women with no previous births^(^ 4 ^)^.

A prospective cohort study, conducted with 110 primiparae, compared the means of PFMS during the pregnancy and after delivery, using perineometry and vaginal digital palpation. These primiparae were followed at four moments: up to 12 weeks of gestation; between 36 and 40 weeks of gestation; between 42 and 60 days after the delivery. The results showed that the PFMS did not change significantly during the pregnancy nor in the puerperium (ANOVA: p =0.78), with a prevalence of weak intensity PFMS. The study also found that there was no statistically significant difference in relation to maternal age, race, marital status, dyspareunia, nutritional status, stool characteristics, type of delivery, perineal conditions, and weight of the newborn^(^ 5 ^)^.

There are several studies on PFMS and genitourinary symptoms from the second half of pregnancy and after childbirth, however, there are few data related to the first trimester. Nevertheless, it is considered important to know the conditions of the pelvic floor (PF) when a woman starts pregnancy because the literature indicates the efficacy of prevention and early treatment of UI, the principle genitourinary symptom, through perineal exercises^(^ 6 ^)^.

The objectives of the present study were: 1. to analyze the PFMS of pregnant women who had one or more vaginal or cesarean deliveries according to age, type of delivery, number of previous vaginal deliveries, perineal conditions, body mass index (BMI), and genitourinary tract symptoms; 2. to compare the PFMS of primigravidae with that of pregnant women who had undergone one or more vaginal or cesarean deliveries.

## Method

Cross-sectional study on the evaluation of PFMS, conducted in five Primary Health Units (PHUs) of the municipality of Itapecerica da Serra, São Paulo state, Brazil, from December 2012 to May 2013.

The study population consisted of pregnant women who met the following inclusion criteria: one or more previous vaginal or caesarean deliveries; up to 12 weeks of gestation; without previous abdominal or urogenital surgery (other than cesarean section); without diseases that could interfere with the PFMS (pelvic organ prolapse, neurological diseases, diabetes, pelvic or spinal injury); without difficulty in communicating due to decreased hearing acuity or impaired speech. Exclusion criteria were: multiple gestation; resistance to vaginal digital palpation or insertion of the perineometer into the vagina.

The sample consisted of 110 pregnant women. This is the same sample size defined in an earlier study of 110 primigravidae with the same inclusion and exclusion criteria^(^ 5 ^)^, which were considered to be the historical controls for comparison with the pregnant women in this study.

Data were collected by two previously trained researchers and all the participants underwent measurement of the PFMS by perineometry and vaginal digital palpation.

It is noteworthy that in the study with the primigravidae (historical control) only one researcher measured the vaginal digital palpation. In the present study, the same researcher was one of the two who performed the PFMS evaluation. However, concordance between the examiners was not analyzed.

To avoid bias in the data, a table was produced for the random application of the sequence of PFMS measurement methods, using a statistical program. Accordingly, the perineometry could be performed first and then the vaginal digital palpation, or vice versa.

For the perineometry, an electronic pressure Peritron^TM^ 9301 model perineometer of *Laborie*, Canada, was used, which records the contractions of the PF muscles through a sensor attached to an 8 centimeters (cm) long and 3 cm diameter silicone vaginal probe. The sensor numerically measures the contraction of the pelvic floor muscles, which can range from 0.1 to 300 cm of water (cmH_2_O). This device does not differentiate between the contractions of the PF and those of the abdominal muscles.

To control for abdominal relaxation during the PFMS measurement, a surface electromyograph, model Bio-ADS1200^(r)^, Lynx, was used, which detects, by means of external electrodes, the electrical activity of the muscle during rest and contraction. The registration of the PFMS indicated by the perineometer was only considered when the electromyograph chart indicated activity of the abdominal musculature compatible with rest (electromyograph scale between 0 and 10 microvolts).

The PFMS evaluated by vaginal digital palpation was graded according to the modified Oxford Scale^(^ 7 ^)^, which considers: Grade 0 - lack of muscle response; Grade 1 - flicker of non-sustained contraction; Grade 2 - presence of low intensity, but sustained, contraction; Grade 3 - medium contraction, felt like an increase in intravaginal pressure, compressing the fingers of the examiner with small cranial elevation of the vaginal wall; Grade 4 - satisfactory contraction, compressing the fingers of the examiner, with elevation of the vaginal wall toward the pubic symphysis; Grade 5 - strong contraction, firm compression of the examiner's fingers with positive movement toward the pubic symphysis.

## Procedures for measuring the PFMS

1. The woman was placed in the gynecological position, with the genital region and the legs bare and protected by a sheet; 2. The four electromyograph electrodes were connected on the abdominal rectus muscles (2 electrodes on the right side and 2 on the left side, between the top edge of the pubis and the umbilicus region); 3. Procedure gloves were put on; 4. The women were taught to perform the contractions as if "holding" the urine, using only the muscles of the AP, avoiding contraction of the abdominal, adductor and gluteal muscles.

## Perineometry

1. The elastic probe was fitted with a non-lubricated disposable condom; 2. The condom was lubricated with water-based gel; 3. The woman was instructed to relax the PFM; 4. The perineometer was connected; 5. Four to six centimeters of the probe was introduced into the vagina; 6. The perineometer was inflated until the scale reached 100 cmH_2_O and the device was zeroed, as recommended by the manufacturer; 7. The woman was asked to perform, and hold for five seconds, the voluntary contraction of the perineal muscles around the vaginal probe, in a sequence of three sessions, with an interval of 30 seconds between each; 8. The vaginal probe remained in place throughout all the PFMS measurements; 9. The PFMS measurements were recorded when the movement of the perineal probe in the cranial direction was observed, indicating that the contraction had been performed. When this was not observed, Grade 0, according to the Oxford Scale, was recorded; 10. The PFMS of the highest pressure was considered; 11. The woman rested for one minute before starting the vaginal digital palpation (if it had not been previously performed, according to the randomization).

## Vaginal digital palpation

1. The two distal phalanges of the index and middle fingers were entered into the vagina, using lubricant gel; 2. The woman was asked to perform, and hold for as long as possible, the voluntary contraction of the perineal muscles around the fingers of the examiner, in a sequence of three sessions, with an interval of 15 seconds between each; 3. The fingers remained in the vaginal throughout all the PFMS measurements; 4. The highest contraction classification according to the Oxford scale was recorded; 5. The woman rested for one minute before starting the perineometry (if it had not been previously performed, according to the randomization).

Descriptive and inferential statistical analysis was performed. For the intergroup analysis (comparison between primigravidae and pregnant women with one or more previous normal or cesarean deliveries), the chi-square test with Monte Carlo simulation was used when indicated. For the intragroup analysis (sample of 110 pregnant women with one or more previous vaginal or cesarean deliveries) Student's t-tests and ANOVA were used. All the tests were performed in the two-tailed fashion, assuming a 5% (p value=0.05) probability of the occurrence of type 1 error. 

The study was approved by the Research Ethics Committee (034/2011/CEP-EACH). The women were enrolled in the study only after receiving information and signing the Terms of Free Prior Informed Consent.

It should be noted that the researchers did not have any link with the manufacturers or distributors of the equipment used in this study.

## Results

The mean age of the women was 28.6 years, with standard deviation (SD) of 5.9, and mean gestational age of 7.8 (SD = 2.2) weeks. The majority were of non-white skin color (70.9%), with 29.1% white. Regarding occupation, 36.5% were employed and 49.5% were housewives. In relation to marital status, 82.7% of the women reported living with a partner and 77.3% had complete high school education, 8.2% incomplete primary education and 4.5% had incomplete higher education. No pregnant women reported regularly performing any type of perineal exercise. Table 1 shows the comparison between the sociodemographic characteristics of the primigravidae (historical control) and the pregnant women with one or more vaginal deliveries. The groups presented heterogeneity with respect to age, marital status and education. Among the pregnant women with previous deliveries, there were more women over 20 years of age, who lived with a partner and who had at least completed high school.

**Figure 1 -:**
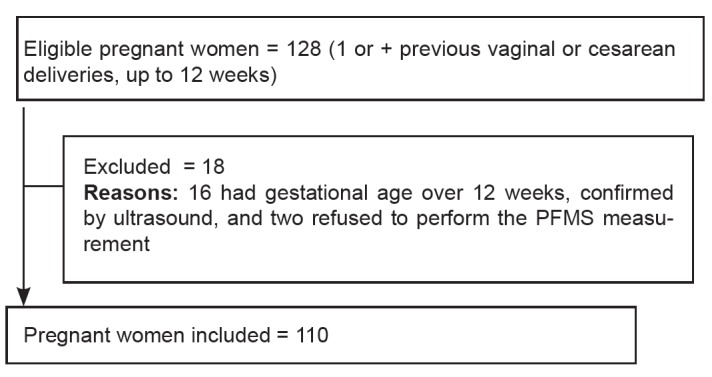
Flowchart of the pregnant women that participated in the study. Itapecerica da Serra, SP, Brazil. December 2012 to May 2013

**Table 1 -:** Number and percentage of women according to sociodemographic characteristics of the primigravidae and of the pregnant women who had undergone one or more vaginal or cesarean deliveries. Itapecerica da Serra, SP, Brazil. December 2012 to May 2013

Variable		Primigravidae			1 or + previous vaginal or cesarean deliveries		
		n	%		n	%	p-value
Age (years)		(n=110)			(n=109)		<0.001*
	14 ├ 20	50	55.5		7	6.4	
	20 ├ 30	49	44.5		59	54.2	
	30 ├ 40	11	10.0		36	33.0	
	≥ 40	-	-		7	6.4	
Skin color		(n=110)			(n=110)		0.76^†^
	Non-white	81	73.6		78	70.0	
	White	29	26.4		32	29.1	
Occupation		(n=110)			(n=107)		0.10^†^
	Paid employment	49	44.5		39	36.5	
	Housewife	39	35.5		53	49.5	
	Student	22	20.0		15	14.0	
Marital status		(n=110)			(n=110)		<0.001^†^
	Lives with partner	67	60.9		91	82.7	
	Does not live with partner	43	39.1		19	17.3	
Education		(n=110)			(n=109)		<0.001*
	None to incomplete high school	51	46.4		20	18.3	
	Complete high school or incomplete higher education	54	49.1		89	81.7	
	Complete higher education	5	4.5		-	-	

* Estimated chi-square (Monte Carlo simulation)

† Chi-square

The PFMS, analyzed according to maternal age, type of delivery in previous pregnancies, number of previous vaginal deliveries, perineal conditions, BMI, urge urinary incontinence (UUI), urinary tract infection, and dyspareunia is presented in Table 2.

**Table 2 -:** Mean of the perineometry and degrees of the vaginal digital palpitation (degrees of the Oxford scale) of the pregnant women that had undergone one or more normal or cesarean deliveries, according to age, type of delivery, previous vaginal deliveries, perineum conditions, BMI, and genitourinary symptoms. Itapecerica da Serra, SP, Brazil. December 2012 to May 2013

Variable		n	Perineometry		Vaginal digital palpation			
					≤ 2	3	≥ 4	p-value
			Mean (SD)	p-value	n (%)	n (%)	n (%)	
Age (years) (n=109)				0.36*				0.58^†^
	14 ├ 20	7	27.6 (12.8)		6 (85.7)	1 (14.3)	-	
	20 ├ 30	59	34.0 (23.2)		47 (79.7)	9 (15.2)	3 (5.1)	
	30 ├ 40	36	30.7 (18.3)		26 (72.3)	7 (19.4)	3 (8.3)	
	≥ 40	7	45.0 (24.0)		4 (57.1)	3 (42.9)	-	
Type of delivery (n=110)				0.80^‡^				0.83^†^
	Vaginal^§^	85	33.0 (20.7)		65 (76.5)	16 (18.8)	4 (4.7)	
	Caesarean	25	34.2 (23.2)		18 (72.0)	5 (20.0)	2 (8.0)	
Previous vaginal deliveries (n=85)				0.13*				0.25^†^
	1	49	32.7 (21.0)		40 (81.6)	7 (14.3)	2 (4.1)	
	2	22	30.6 (20.0)		18 (81.9)	3 (13.6)	1 (4.5)	
	3	10	45.0 (18.8)		5 (50.0)	4 (40.0)	1 (10.0)	
	4 or more	4	18.9 (11.2)		2 (50.0)	2 (50.0)	-	
Perineum conditions (n=85)				0.04^‡^				0.26^†^
	With episiotomy	55	36.2 (22.7)		39 (70.9)	13 (20.6)	3 (5.5)	
	Without episiotomy	30	26.9 (15.0)		26 (86.7)	3 (10.0)	1 (3.3)	
BMI (n=110)				0.49*				0.66^†^
	Underweight	11	35.6 (10.9)		6 (54.5)	4 (36.4)	1 (9.1)	
	Appropriate weight	41	36.8 (24.3)		32 (78.0)	7 (17.1)	2 (4.9)	
	Overweight	41	30.5 (22.8)		31 (75.6)	7 (17.1)	3 (7.3)	
	Obese	17	29.7 (11.9)		14 (82.4)	3 (17.6)	-	
UUI (n=110)				0.10^‡^				0.41^†^
	Yes	22	29.0 (15.4)		19 (86.4)	2 (9.1)	1 (4.5)	
	No	88	34.3 (22.4)		64 (72.7)	19 (21.6)	5 (5.7)	
Urinary tract infection (n=110)				0.37^‡^				0.52^†^
	Yes	45	32.4 (20.8)		36 (80.1)	8 (17.7)	1 (2.2)	
	No	65	33.8 (21.6)		47 (72.3)	13 (20.0)	5 (7.7)	
Dyspareunia (n=110)				0.13^‡^				0.57^†^
	Yes	29	30.0 (15.6)		24 (82.8)	4 (13.8)	1 (3.4)	
	No	81	34.4 (22.9)		59 (72.9)	17 (21.0)	5 (6.1)	

* ANOVA

† Estimated chi-square (Monte Carlo simulation)

‡ Student's t test

§ 9 women with previous cesarean

The perineometry showed a mean of 33.4 (SD = 21.2) cmH_2_O. The mean PFMS was higher among women aged 40 years or more (45.0; SD = 24.0 cmH_2_O). Among the pregnant women who had had previous vaginal deliveries, the mean PFMS was 33.0 (SD = 20.7) cmH_2_O (min = 4.4; max = 107.0) and among those who had had cesarean deliveries the mean was 34.2 (SD = 23.2) cmH_2_O (min = 8.2; max = 105.9). Regarding the number of previous vaginal deliveries, the mean PFMS was lower (18.9; SD = 11.2 cmH_2_O) among the pregnant women who had undergone four or more deliveries. For the others, those with three previous normal deliveries presented a mean PFMS of 45.0 (SD = 18.8 cmH_2_O), higher than those with one or two previous normal deliveries (32.7; SD = 21.0 and 30.6; SD = 20.0 cmH_2_O, respectively). However, none of the differences were significant.

Regarding BMI, the pregnant women who were the correct weight had slightly higher PFMS (36.8; SD = 24.3 cmH_2_O) than the other pregnant women. In relation to genitourinary tract symptoms, the women with UUI (20.0%), urinary tract infection (40.9%), and dyspareunia (26.4%) presented slightly lower PFMS than those without complaints. There was a statistically significant difference in the PFMS between the women who had undergone an episiotomy and those who had not (36.2; SD = 22.7 *vs.* 26.9; SD = 15.0).

In the vaginal digital palpation, none of the variables analyzed showed a statistically significant difference. It is noteworthy that there was no grade ≥ 4 among the women in the extreme age groups, those with four or more deliveries or those with obesity. Accordingly, the proportion of pregnant women with grade ≥ 4 was higher among those without genitourinary tract symptoms, compared to those who reported these symptoms. Although not statistically significant, the women who had undergone episiotomies had higher grades in the Oxford scale, considering the degrees above 2 (26.1% with episiotomy *vs*. 13.3%, without).

As shown in Table 3, among the women with previous normal or cesarean deliveries, 75.4% presented grade ≤ 2 in the vaginal digital palpation and only 5.5% presented grade ≥ 4. Conversely, more than half of the primigravidae presented grade ≥ 4, with a statistically significant difference in relation to parity. It should be noted that in the study with the primigravidae only one researcher measured the vaginal digital palpation and in the current study, the same researcher of the previous study and another researcher performed the measurements, however, it was not ascertained whether there was concordance between the examiners.

**Table 3 -:** Vaginal digital palpation (degrees of the Oxford scale) in primigravidae and women who had undergone one or more previous vaginal or cesarean deliveries, with up to 12 weeks of gestation. Itapecerica da Serra, SP, Brazil. December 2012 to May 2013

Vaginal digital palpation	Primigravidae*		1 or + previous vaginal or cesarean deliveries		p-value
	n	%	n	%	
≤ 2	34	39.9	83	75.4	<0.001^†^
3	20	18.2	21	19.1	
≥ 4	56	50.9	6	5.5	
Total	110	100	110	100	

* Historical control(5)

† Chi-square

## Discussion

Among the sample of women studied, the PFMS, evaluated by vaginal digital palpation, corroborates the findings of other studies with pregnant women, indicating that, from the first trimester, many pregnant women have weak contraction of the PF muscles (Oxford scale ≤ 2)^(^ 5 ^)^.

In the present study, this PFM weakness was more frequent among women with one or more previous cesarean or vaginal deliveries, compared with the primigravidae historical control^(^ 5 ^)^, reinforcing the impact in the PF muscles caused by pregnancy and delivery.

Regarding the perineometry, the interpretation and comparison with other studies may be hampered by the diversity of equipment and methods used in the evaluation^(^ 8 ^-^ 12 ^)^. For this reason, the perineometry values obtained for the historical controls^(^ 5 ^)^ could not be used in this study due to the use of a Perina 996-2^(r)^ perineometer, with a scale ranging from 1.6 to 46.4 mmHg and a mean obtained of 15.9 mmHg among 110 primigravidae in the first trimester of pregnancy^(^ 5 ^)^; this corresponds to a weak contraction^(^ 8 ^)^.

As stated in the method, the perineometer adopted in this study was the Peritron^TM^ 9300, with a scale ranging from 0.1 to 300 cmH_2_O and the PFMS mean obtained among pregnant women who had previous vaginal or cesarean delivery was 33.4 (SD = 21.2) cmH_2_O. 

The PFMS means, evaluated by the perineometer were similar among pregnant women with a history of vaginal or cesarean delivery (33.0 and 34.2 cmH_2_O, respectively). However, it was not possible to classify this PFMS as weak, normal or strong, because no parameters have been defined in the literature for the device used. 

A study conducted with 30 nulliparae and 64 primiparae (32 post-vaginal and 32 post-cesarean delivery), between four and six months after the delivery, examined the relationship between the type of delivery and the PFMS, using the perineometry and vaginal digital palpation methods. The authors concluded that the PFMS was lower among women who had undergone vaginal delivery, and was also decreased among those who had undergone cesarean section, compared to the nulliparous women. Furthermore, higher parity and vaginal delivery have been considered predictors of UI^(^ 8 ^)^. These results are similar to those of the present study, as although there was no statistically significant difference in PFMS among the women with a history of vaginal or cesarean delivery, the mean of the perineometry was lower among those with a history of vaginal deliveries.

The study aimed to investigate the obstetric, neonatal and clinical predictors for UUI and used the perineometer of this study showing that a PFMS ≤ 35.5 cmH_2_O was the strongest predictor of UUI ^(^ 13 ^)^.

Another study that evaluated genitourinary tract symptoms in 120 women three years after childbirth concluded that the pregnancy is more related to UUI than the delivery^(^ 14 ^)^. Conversely, these genitourinary tract symptoms can be prevented or improved through strengthening of the PFM through exercises^(^ 15 ^)^.

The women in the present study were in the first trimester of pregnancy and still had a high frequency of genitourinary tract symptoms i.e., UUI, urinary tract infection, and dyspareunia. This finding is relevant because, with the exception of urinary tract infections, the diagnosis and treatment of which integrate prenatal care protocols, in general, pregnant women are not asked to report the other symptoms.

Regarding the PFMS and genitourinary tract symptoms, maternal age and BMI showed no statistically significant difference, as in other studies^(^ 5 ^,^ 10 ^,^ 15 ^)^. However, researchers have found that these variables can have an impact on the PFMS^(^ 16 ^-^ 17 ^)^. Urinary and anal incontinence may be associated with damage to the PF muscles caused by vaginal delivery^(18).^

A study that evaluated UI, comparing vaginal with cesarean delivery, found fewer women with urinary incontinence after cesarean delivery, compared with women who underwent vaginal delivery. However, this difference associated with the type of delivery was transient, as three months later it was not significant^(^ 19 ^)^.

A study carried out with 15,307 women concluded that those who had undergone a previous caesarean section had a higher prevalence of UI that the nulliparous women (15.9% *vs.* 10.1%, respectively). The authors considered that the bladder catheter or possible difficulties in fetal extraction during the cesarean section can lead to injury to the bladder mucosa and, consequently, the occurrence of genitourinary tract symptoms^(^ 20 ^)^.

Another study^(^ 21 ^)^ found a higher prevalence of UI in women who had undergone vaginal delivery (21.0%), probably related to the lack of preparation of the perineum before or during pregnancy, perineal trauma sustained during the delivery, inadequate repair of the perineum, and the lack of monitoring after the delivery. The researchers suggest that a larger study should be performed to evaluate these variables, as, although the results did not show a statistically significant difference, there was a decrease in PFMS among the women who had genitourinary tract symptoms or were obese.

In relation to the perineum conditions, when the PFMS was evaluated by the vaginal digital palpitation, no statistical significant difference was observed; however, when evaluated by perineometry, the pregnant women who had undergone a previous episiotomy had greater PFMS than those that had never undergone an episiotomy, with a statistically significant difference. These results differ from those obtained in other studies as well as those from the systematic review of six randomized controlled trials, as their results showed that the restricted use of episiotomy reduces morbidity of the PF muscles^(^ 22 ^-^ 23 ^)^.

In the present study, it is should be noted that the population was young, of reproductive age without hormonal changes caused by the menopause or other adverse effects of advanced age on the PF muscles. However, according to the vaginal digital palpation, the PFMS was below that expected and considered normal and strong (grades 4 and 5 of the Oxford scale) ^(^ 7 ^-^ 8 ^)^.

There are several factors that can influence the PFMS and, although the decrease that occurs in every pregnancy or delivery is not always significant, over several pregnancies and deliveries, this decrease can become significant^(^ 5 ^)^. 

Accordingly, it is important that genitourinary tract symptoms are considered during the prenatal and postpartum periods and that, with the occurrence of morbidities, monitoring and effective treatment is performed, so that this will not interfere in the choice of delivery method and that women are not discouraged from becoming pregnant. 

Several studies have shown that perineal exercises during pregnancy are a safe and effective method for maintaining urinary continence, both for women with a history of UI, as well as for those without urinary leakage^(^ 14 ^,^ 24 ^)^.

There was good acceptance by the pregnant women to participate in the study considering that, although the measurement of PFMS is not a painful procedure, it can cause embarrassment and discomfort. They were guided and encouraged to perform exercises to strengthen the PF muscles.

Regarding the perineum conditions during delivery i.e., episiotomy, spontaneous lacerations or tissue integrity, prospective studies, with monitoring of the women from before the first pregnancy until a long period after the birth, could help to evaluate its real impact on the PF muscles.

In relation to the limitations of this study, it should be noted that the variation observed in the perineometry mean, with a large standard deviation, the measurement of PFMS performed by different examiners, coupled with the lack of methodological standardization among the different studies made it difficult to interpret and compare the PFMS values. The establishment of a PFMS profile during and after the pregnancy remains a challenge for further studies.

## Conclusion

The PFMS of the majority of the pregnant women was classified as weak in the first trimester of the pregnancy. 

The decrease in PFMS was not associated with maternal age, delivery method, number of previous vaginal deliveries, BMI, UUI, urinary tract infection, or dyspareunia. The PFMS was significantly higher among the women who had undergone episiotomies, compared to those who never experienced the intervention. The PFMS, when evaluated by vaginal digital palpation, presented a statistically significant lower degree among women who had undergone one or more previous deliveries, both vaginal and cesarean, compared with the primigravidae of the study, adopted as the historical control.
